# Air pollution, cardiorespiratory fitness and biomarkers of oxidative status and inflammation in the 4HAIE study

**DOI:** 10.1038/s41598-024-60388-w

**Published:** 2024-04-26

**Authors:** Lukas Cipryan, Martina Litschmannova, Tomas Barot, Tomas Dostal, Dominik Sindler, Petr Kutac, Daniel Jandacka, Peter Hofmann

**Affiliations:** 1 Department of Human Movement Studies and Human Motion Diagnostic Centre, The University of Ostrava, Ostrava, Czech Republic; 2 Department of Applied Mathematics, Faculty of Electrical Engineering and Computer Science, VSB – Technical University of Ostrava, Ostrava, Czech Republic; 3 Department of Mathematics with Didactics, The University of Ostrava, Ostrava, Czech Republic; 4 Institute of Human Movement Science, Sport and Health, University of Graz, Graz, Austria

**Keywords:** Air pollution, Oxidative status, Inflammation, $\dot{V}{\text{O}}_{{2{\text{peak}}}}$, Trunk fat mass, Diseases, Risk factors

## Abstract

The aim of this study was to investigate the associations between cardiorespiratory fitness (CRF), long-term air pollution exposure and biochemical markers of oxidative status and inflammation. This is a cross-sectional investigation focusing on biochemical markers of oxidative status and inflammation. Participants were Caucasian (N = 1188; age 18–65 years) who lived for at least 5 years in a high air-polluted (Moravian-Silesian; MS) or low air-polluted (South Bohemia; SB) region of the Czech Republic. Healthy runners and inactive individuals were recruited. A multiple regression analysis was used to explain the relationship between multiple independent variables (CRF, trunk fat mass, sex, socioeconomic status, and region (MS region vs. SB region) and dependent variables (oxidative status, inflammation). CRF, trunk fat mass, age and sex significantly predicted almost all selected markers of oxidative status and inflammation (except GSSG, GSH/GSSG and BDNF). Participants living in the MS region presented significantly higher GPx (by 3.1%) and lower BDNF values (by 4.5%). All other investigated biochemical markers were not significantly influenced by region. We did not find meaningful interactions between long-term air-pollution exposure versus markers of oxidative status and inflammation. However, we showed various significant interactions with sex, age, CRF and body composition. The significant association of living in the high air polluted MS region with the BDNF level warrants further attention.

## Introduction

Air pollution (AP) is recognized as the single biggest environmental threat to human health^1^. It has been demonstrated that 9 out of 10 people worldwide live in places where air quality exceeds WHO guideline limits. 4.2 million premature deaths every year occur as a result of the exposure to ambient AP, mostly in low-to-middle-income countries^2^. A considerable proportion of premature deaths could still be avoided annually also in European areas by lowering air pollution levels^3^, even if AP, AP-related morbidity and mortality have decreased substantially in Europe in the last three decades^4^.

AP is a complex mixture of gases (e.g. ozone, nitrogen and sulfur oxides), volatile organic compounds (e.g. polycyclic aromatic hydrocarbon) and particulate matter. Especially, fine particulate matter smaller than 2.5 µm (PM_2.5_) is one of the principal air pollutants attracting most scientific and regulatory attention. PM_2.5_ is considered a leading cause of global morbidity and mortality as it promotes the development of respiratory diseases as well as chronic cardiometabolic conditions including atherosclerosis, hypertension, and diabetes mellitus^5^. In addition, AP has been identified as a contributing factor in inducing epigenetic alterations, along with respiratory, neurological, psychiatric, and musculoskeletal diseases^6,7^. Furthermore, the cardiorespiratory system is significantly affected not only by a long-term, but also short-term ambient AP exposure^8^. Thus, AP should be considered as one of several major modifiable risk factors in the prevention and management of cardiovascular disease^9^.

The mechanistic clarification of the detrimental AP effect on morbidity and mortality is mediated by oxidative stress and an inflammatory response that affects organ function^10–12^, as well as genetic and epigenetic pathways^10^. The development of chronic cardiovascular disease can be induced via three potential pathways: a “spill over” of pro-inflammatory or oxidative stress mediators into the systemic circulation generated in the lungs, the triggering of autonomic nervous system imbalance, and the penetration of particles (ultrafine particles PM_0.1_) or components (soluble metals, organic compounds) directly into cardiovascular tissues^13^. Each pathway alone or altogether can elicit a host of adverse responses depending upon the dose and time course of exposure as well as the individual level of susceptibility^5^. These three factors can lead to atheroma progression, endothelial dysfunction, impaired fibrinolysis, platelet hyperreactivity and possibly arrhythmogenesis^9^.

Regular physical activity and exercise are known to be beneficial to health. However, the most accessible forms of exercise, such as walking, running and cycling are often performed outdoors, which means an increased exposure to environmental AP specifically in urban regions with a high density in traffic and industry^14,15^. An increased risk of cardiorespiratory function, immune function and exercise performance disruption has been associated with the combination of AP and exercise^16^. Moreover, increased ventilation during exercise causes a greater influx of air and pollutants into the airways, and those might even reach systemic circulation^17,18^. A 12-week aerobic training program in an urban environment with high traffic-related air pollution exposure versus training in a rural environment with lower traffic-related AP increased markers of respiratory and systemic inflammation (leukocytes count, neutrophil counts, exhaled nitric oxide). These changes positively correlated with personal PM_0.1_ exposure during training^19^. Even a short-20-min-duration of exercise near a busy roadway was sufficient to significantly increase blood levels of volatile organic compounds (toluene, ethylbenzene and xylene)^20^. Furthermore, 5 days of aerobic outdoor exercise significantly impaired nasal mucociliary clearance in a street runners group when compared with a forest runners group^21^. This suggests that regular outdoor exercise in AP can be potentially harmful to cardiorespiratory health. Therefore, reducing exercising outdoors during peak exposure times (rush hours) has been recommended to reduce exposures or susceptibility to AP^5,22^. However, practicing physical activity in air polluted environments might be more favorable to the health of older adults than remaining in sedentary behaviour^23^. Some molecular mechanisms have been recently described, e.g. linking AP to bone damage^24^, atopic dermatitis^25^, epigenetic modifications^26^, asthma in children^27^, gestational diabetes^28^, adverse cardiac remodelling^29^ among many others, although the main risks have not been identified yet. However, the combined effect of AP and exercise is an emerging research topic with still unknown molecular mechanisms^30^. Therefore, the aim of this study was to investigate the associations between cardiorespiratory fitness (CRF), long-term air pollution exposure and biochemical markers of oxidative (redox) status and inflammation using a multivariate regression analysis in a large cohort of healthy participants.

## Results

### Study population

The detailed characteristics of the participants are presented in Table 1. The presented results of the multivariate median regression analysis (Model 1) included 1188 participants who performed the graded exercise test (GXT) for CRF and peak oxygen consumption ($\dot{V}{\text{O}}_{{2{\text{peak}}}}$) assessment. 116 participants out of 1314 were excluded from the statistical analysis because they did not meet the preliminary health conditions requirements for the GXT (mostly because of high blood pressure). Other 10 participants were excluded because of missing data of various kind. The descriptive characteristics of this selective cohort are summarized in Tables 1 and 2.

**Table 1:** Basic characteristics of the cohort (Model 1; N = 1188).

Males/females	635 (53%)/553 (47%)	
MS/SB region	663 (56%)/525 (44%)	
SES-1	62 (5%)	
SES-2	610 (51%)	
SES-3	516 (44%)	

	(Min; Max)	M (IQR)
Age (years)	(18.0; 65.0)	38.0 (27.0; 46.0)
Height (cm)	(148.5; 201.6)	174.5 (167.8; 181.4)
Body mass (kg)	(40.5; 127.4)	74.0 (63.8; 84.3)
Total body fat (%)	(3.5; 50.5)	20.6 (15.2; 27.7)
Trunk fat mass (%)	(14.1; 51.9)	26.9 (22.2; 32.4)
$\dot{V}{\text{O}}_{{2{\text{peak}}}}$ (ml/kg/min)	(14.6; 70.9)	41.7 (34.5; 49.1)
Systolic BP (mmHg)	(84.0; 180.0)	126.0 (116.0; 134.0)
Diastolic BP (mmHg)	(45.3; 111.7)	78.0 (72.0; 84.3)
Glucose (mmol/l)	(1.65; 8.06)	4.75 (4.48; 5.06)
HbA1c (mmol/l)	(9.0; 63.0)	35.0 (32.0; 37.0)
Total cholesterol (mmol/l)	(1.20; 8.90)	2.90 (2.40; 3.70)
HDL-CH (mmol/l)	(0.68; 3.80)	1.56 (1.27; 1.86)
LDL-CH (mmol/l)	(0.47; 6.39)	2.77 (2.27; 3.41)
Triglycerides (mmol/l)	(0.18; 6.11)	0.98 (0.73; 1.33)
Free fatty acids (mmol/l)	(0.08; 1.44)	0.40 (0.29; 0.56)

SB, South Bohemia Region (low air polluted area); MS, Moravian-Silesian Region (high air polluted area); $\dot{V}{\text{O}}_{{2{\text{peak}}}}$, Peak oxygen consumption; BP, Blood pressure; HbA1c, Glycated hemoglobin; HDL-CH/LDL-CH, High/low density lipoprotein cholesterol; SES, Socioeconomic status represented as an achieved education level (see “Methods” section); M, Median; IQR, Interquartile range.

**Table 2:** Descriptive characteristics of oxidative status, inflammation and other biochemical markers (Model 1).

	(Min; Max)	M (IQR)
SOD (U/ml)	(123.0; 529.0)	237.0 (215.0; 260.0)
GPx (U/l)	(2852; 27,393)	11,221 (9615; 12,878)
GSSG (μmol/l)	(6.0; 228.0)	72.0 (50.0; 97.0)
GSH (μmol/l)	(374; 2610)	1106 (959; 1243)
GSH/GSSG (–)	(2.6; 225.5)	15.5 (10.6; 23.8)
IL-1β (pg/ml)	(0.01; 63.17)	0.39 (0.19; 0.51)
IL-1RA (pg/ml)	(0; 6235)	474 (350; 624)
hs-IL-6 (pg/ml)	(0.00; 145.77)	0.17 (0.07; 0.54)
IL-10 (pg/ml)	(0.01; 64.47)	0.12 (0.05; 0.41)
TNF-α (pg/ml)	(0.02; 10.89)	0.90 (0.38; 1.55)
CRP (mg/l)	(0.50; 55.10)	0.50 (0.50; 1.70)
Fibrinogen (g/l)	(0.45; 5.00)	2.60 (2.29; 2.95)
Adiponectin (μg/ml)	(0.0; 106.19)	5.96 (3.73; 8.96)
Leptin (ng/ml)	(0.0; 92.03)	6.10 (2.39; 12.25)
Adpn/Lep (–)	(0.00; 8570.14)	0.99 (0.41; 2.58)
BDNF (ng/ml)	(0.0; 142.79)	24.15 (18.88; 29.77)

SOD, Superoxide dismutase; GPx, Glutathione peroxidase; GSSG, Glutathione disulphide; GSH, Glutathione; IL-1β, Interleukin 1β; IL-1RA, Interleukin-1 receptor antagonist; hs-IL-6, High-sensitive interleukin 6. IL-10, Interleukin 10; TNF-α, Tumor necrosis factor alpha; CRP, C-reactive protein; Adpn/Lep, Adiponectin-leptin ratio; BDNF, Brain-derived neurotrophic factor; M, Median; IQR, Interquartile range.

### Regional air pollution distribution

**Figure 1:**
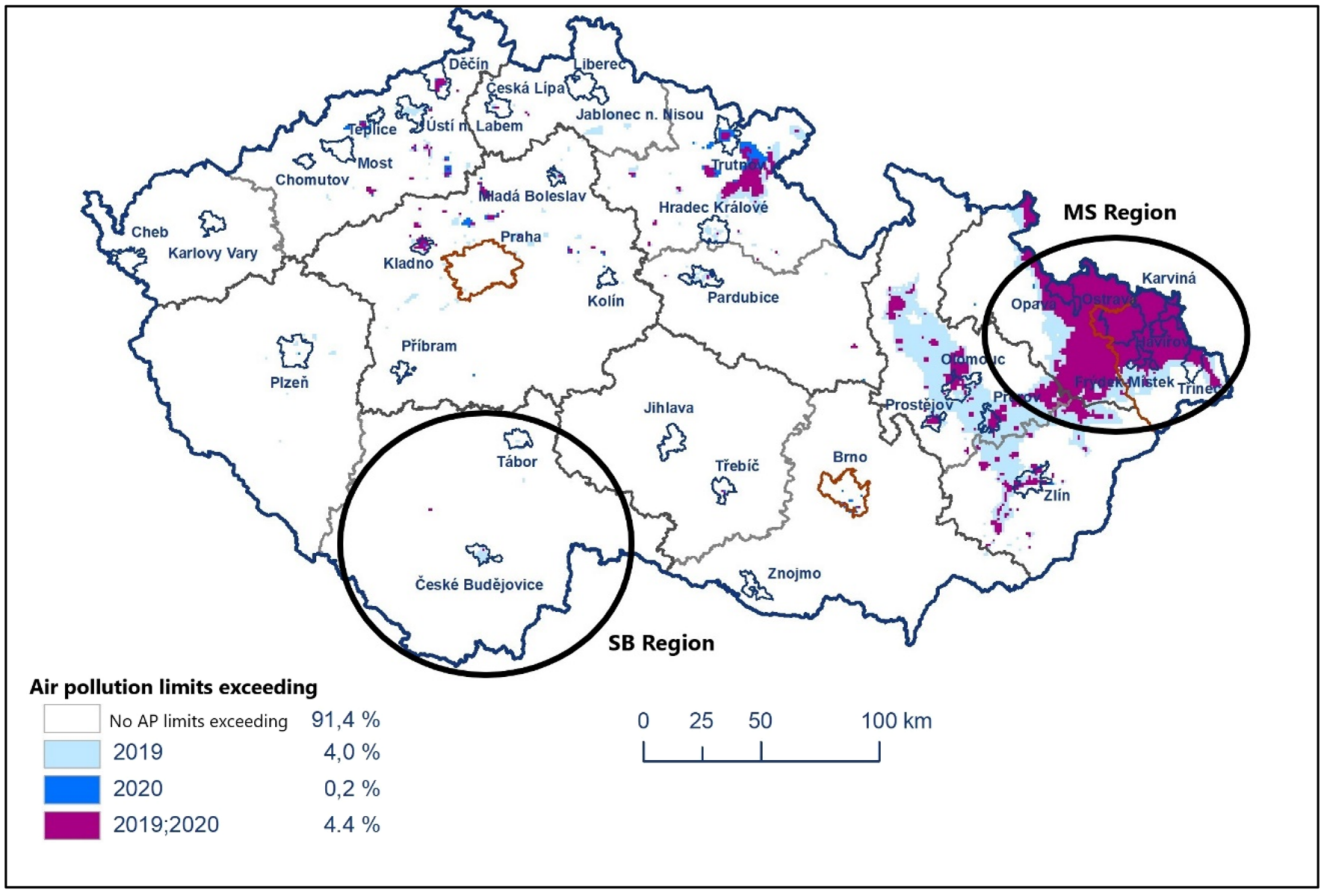
The areas with air pollution limits exceeding without ozone in 2019 and 2020 (source: Czech Hydrometeorological Institute).

The AP in the Czech Republic is getting slightly better during the last monitored period since 2010. However, benzo[a]pyrene (BaP), the particle matters PM_2.5_ and PM_10_ and ozone are the main issues of the AP in the Czech Republic with substantial between region differences^31^. The Moravian-Silesian (MS) region ranks among the long-term most air polluted areas in the Czech Republic whereas the South Bohemia (SB) region is characterized by a low AP load (Fig. 1). For example, the area percentage of total region area with an annual average of the PM_2.5_ and BaP in 2020 higher than a set AP limit of 20 μg m^−3^ and 1 ng m^−3^, respectively, were up to 1.32% and 0% for PM_2.5_ and up to 56.17% and 0.03% for BaP in the MS versus SB region. 94% of the citizens of the specific core urban area of the MS region is exposed to exceeding AP limits frequently^32^.

### Biochemical markers of oxidative status, inflammation and other risk markers

The results of the multivariate median regression analysis (Model 1) for oxidative status, inflammation and other risk markers are presented in the Tables 3, 4 and 5, respectively. Factors significantly related to increased oxidative status markers were male sex (superoxide dismutase (SOD), glutathione (GSH)), MS region (glutathione peroxidase (GPx)), age (GPx) and $\dot{V}{\text{O}}_{{2{\text{peak}}}}$ (GSH). Factors that were significantly inversely related to oxidative status markers included age (SOD) and $\dot{V}{\text{O}}_{{2{\text{peak}}}}$ (SOD). Living in a high or low AP region of the Czech Republic was no significant predictor of oxidative status markers except GPx.

**Table 3:** A multi-regression analyses of the oxidative status markers (Model 1).

	SOD	GPx	GSSG	GSH	GSH/GSSG
(Intercept)	281.269**	10,254.390**	50.981**	977.749**	21.547**
Sex_male	41.881**	203.533	− 3.233	46.132*	1.247
SES 2	0.963	398.488	6.004	− 6.028	− 2.055
SES 3	− 1.688	369.108	5.930	− 21.016	− 2.575
MS Region	0.093	299.622*	− 2.659	− 17.815	0.323
Age	− 0.502**	13.797*	0.191	0.545	− 0.050
$\dot{V}{\text{O}}_{{2{\text{peak}}}}$	− 1.125**	− 7.063	0.275	2.439*	− 0.065

SOD, Superoxide dismutase; GPx, Glutathione peroxidase; GSSG, Glutathione disulphide; GSH, Glutathione; SES, Socioeconomic status represented as an achieved education level (see “Methods” section); MS, Moravian-Silesian Region; $\dot{V}{\text{O}}_{{2{\text{peak}}}}$, Peak oxygen consumption.

*p < .05; **p < .01.

**Table 4:** A multi-regression analyses of the inflammation markers (Model 1).

	IL-1β	IL-1RA	hs-IL-6	IL-10	TNF-α
(Intercept)	− 0.047	733.786**	− 0.085	0.004	1.358**
Sex_male	− 0.036	6.403	− 0.036	− 0.001	0.247**
SES 2	0.055	24.688	0.035	0.003	− 0.105
SES 3	0.050	11.770	− 0.041	− 0.002	− 0.126
MS Region	0.008	− 18.321	0.014	0.006	0.042
Age	0.003**	− 0.577	0.005**	0.001**	− 0.002
$\dot{V}{\text{O}}_{{2{\text{peak}}}}$	0.007**	− 5.921**	0.003	0.002**	− 0.010*

IL-1β, Interleukin 1β; IL-1RA, Interleukin-1 receptor agonist; hs-IL-6, High-sensitive interleukin 6; IL-10, Interleukin 10; TNF-α, Tumor necrosis factor alpha; SES, Socioeconomic status represented as an achieved education level (see Methods); MS, Moravian-Silesian Region; $\dot{V}{\text{O}}_{{2{\text{peak}}}}$, Peak oxygen consumption.

*p < .05; **p < .01.

**Table 5:** A multi-regression analyses of the inflammation and other biochemical markers (Model 1).

	CRP	Fibrinogen	Adiponectin	Leptin	Adpn/Lep	BDNF
(Intercept)	2.099**	3.186**	2141.009*	28,934.969**	− 3.708**	27,082.821**
Sex_male	0.157**	− 0.087*	− 2127.432**	− 3357.052**	0.164*	− 782.848
SES 2	− 0.070	− 0.149*	1097.808	− 426.792	0.339*	− 912.646
SES 3	− 0.151	− 0.203**	1173.761*	− 548.528	0.234	− 1046.872
MS Region	− 0.070	− 0.040	− 261.750	− 578.234	0.108	− 1387.843*
Age	0.001	0.008**	27.874*	− 70.425**	0.025**	16.405
$\dot{V}{\text{O}}_{{2{\text{peak}}}}$	− 0.030**	− 0.015**	71.813**	− 385.193**	0.090**	− 31.878

CRP, C-reactive protein; BDNF, Brain-derived neurotrophic factor; SES, Socioeconomic status represented as an achieved education level (see “Methods” section); MS, Moravian-Silesian Region; $\dot{V}{\text{O}}_{{2{\text{peak}}}}$, Peak oxygen consumption.

*p < .05; **p < .01.

The most frequent significant predictors of inflammatory and other biochemical risk markers were sex, age and $\dot{V}{\text{O}}_{{2{\text{peak}}}}$. Socioeconomic status was significantly associated with fibrinogen, adiponectin and adiponectin/leptin (Adpn/Lep) ratio with beneficial patterns in participants with secondary or higher education when compared to participants with basic education. Living in a high or low AP region of the Czech Republic was a significant predictor for brain-derived neurotrophic factor (BDNF) (M (IQR) for MS and SB Regions: 23.7 (18.6; 29.3) ng/ml and 24.8 (19.5; 30.3) ng/ml) with lower values in the Moravian-Silesian (high AP) Region (Tables 5, S6). Of note, BDNF was significantly predicted also by trunk fat mas (%) (Model 2, Table S5).

### Sensitivity analysis

We revealed a significant linear correlation between $\dot{V}{\text{O}}_{{2{\text{peak}}}}$ as a measure of CRF and trunk fat mass (%) (Spearman´s ρ (95% CI): − 0.813 (− 0.832, − 0.793), *p* < .001). This collinearity does not allow to analyse both variables within one regression model. However, we used this association for building an alternative Model 2 with trunk fat mass (%) instead of $\dot{V}{\text{O}}_{{2{\text{peak}}}}$. Therefore, we also present the multivariate median regression model of this remodelled cohort (Model 2, N = 1300, Supplemental Material) for checking the robustness of the Model 1 results. The sensitivity analysis revealed minor changes of the results when $\dot{V}{\text{O}}_{{2{\text{peak}}}}$ was replaced by trunk fat mass (%) in the statistical model. Nevertheless, unlike in Model 1, Model 2 showed that leptin and BDNF were significantly lower in the MS Region (Tables S3–S5).

## Discussion

The presented cross-sectional study primarily investigated the associations between long-term air-pollution exposure (i.e. living in a low or high AP region of the Czech Republic), CRF and biochemical markers of oxidative status, inflammation and other biochemical risk factors by using a multivariate median regression analysis. The covariates sex, age, trunk fat mass (%) and socioeconomic status were included also into the multi-regression analyses. We showed significant interactions between the group of these potential predictors and dependent biochemical variables. CRF (and trunk fat mass within the alternative statistical model), age and sex (separately or in their various combinations) significantly predicted almost all selected markers of oxidative status and inflammation (except glutathione disulphide (GSSG), GSH/GSSG and BDNF). Participants living in the MS region with high ambient AP presented significantly higher GPx (by 3.1%) and lower BDNF values (by 4.5%). All other investigated biochemical markers were not significantly influenced by region. However, we must highlight the importance of CRF and body composition as modifiable determinants of health.

### Oxidative status biochemical markers

GPx activity was the only variable which was significantly higher in the high air polluted MS Region. It also increased significantly with higher age. The GPx protein family is one of the main enzymes that protects cells from oxidative stress and maintains redox balance. GPx works with SOD, catalase, GSH and others to form a whole enzymatic antioxidant system, reduces reactive oxygen species and limits their toxicity^33^. Therefore, we would expect to see comprehensive alterations also of the other oxidative status variables in participants living in the high air polluted MS region due to an excessive oxidative stress compared to living in the low AP SB region. As GPx modulates the balance between GSH and GSSG, a decrease of the GSH/GSSG ratio indicates a shift of the redox balance toward oxidizing conditions^34^. However, we found no significant GSH/GSSG ratio association with any presented predictor. Of note, the absolute between region GPx differences are suggested not meaningful (MS and SB Region; M (IQR): 11,348 (9579; 12,967) U/l and 11,000 (9629; 12,801) U/l, respectively) (Table S6). Nevertheless, oxidative stress is considered a primary pathway connecting AP exposure and chronic non-communicable diseases. Living in high pollution areas may have larger effects on health than short term day-to-day variations in air pollution^35^. Therefore, we cannot exclude that even this isolated and relatively small 3.1% between region median difference in GPx from a single time point analysis may pose an important sign of higher oxidative stress caused by long-term air pollution exposure.

$\dot{V}{\text{O}}_{{2{\text{peak}}}}$ was significantly associated with reduced SOD activity and increased GSH levels but it was not accompanied by an alteration of the GSH/GSSG ratio. An increased activity of these key enzymes involved in the complex antioxidant defence may indicate a compensatory response to the oxidizing conditions^36^. However, the presented findings are inconsistent to make an unequivocal conclusion about a potential oxidative stress or changes in antioxidant capacity.

### Biochemical markers of inflammation

Participants from the high AP MS region presented significantly lower BDNF when compared to the participants from the low AP SB region. Neurotrophins, such as BDNF, promote the development, health and survival of neurons. BDNF levels are reduced in a number of neurodegenerative disorders, including Alzheimer and Parkinson disease^37^ and can be a biomarker of major depressive disorder and antidepressant response^38^. While exercise increases the BDNF level and enhances cognitive function^39^, this exercise-induced effect might be eliminated by air pollution exposure^40^. In contrary, Silveira et al. (2022) showed that 50-km cycling in traffic-induced air pollution provoked a greater acute exercise-induced increase in BDNF levels when compared to filtered air conditions^41^. Despite these diverse findings about the combined effect of AP and exercise the significantly lower BDNF levels (by 4.3%) in the high air polluted region in the Czech Republic when compared to low air polluted region deserves further research attention. A prospective research study focusing on clinical endpoints of neurodegenerative diseases is needed to assess an importance of the presented BDNF findings.

We showed the beneficial role of regular exercise, expressed indirectly via $\dot{V}{\text{O}}_{{2{\text{peak}}}}$ as a measure of cardiorespiratory fitness (CRF) (in the presented study. An increased $\dot{V}{\text{O}}_{{2{\text{peak}}}}$ was significantly associated with anti-inflammatory cytokine interleukin-10 (IL-10), adiponectin and Adpn/Lep ratio and inversely associated with health risk markers such as tumour necrosis factor alpha (TNF-α), C-reactive protein (CRP), fibrinogen and leptin. Most of these variables were also significantly associated with sex and age but were not related to living in a high and low AP region even if literature indicates some relationships with AP. For example, CRP is an acute phase reactant secreted mainly by the liver and released in high concentrations in blood after tissue injury or infection. It is widely used in clinical practice as a marker of infection and/or inflammation but there is also an increased evidence that some forms of CRP can have pro-inflammatory and pro-thrombotic properties^42^. An elevated circulating CRP has been found to be strongly associated with a long-term, unlike short-term, exposure to AP^43^ and this association has been found even in settings with low to moderate AP levels^44^. Even if there were some CRP outliers in both study regions, the medians remained fully within the physiological range, i.e. up to 3 mg/L^45^ (MS and SB Region; M (IQR): 0.50 (0.50; 1.60) mg/l and 0.50 (0.50; 1.90) mg/l, respectively) (Table S6).

Similarly to CRP, an increased $\dot{V}{\text{O}}_{{2{\text{peak}}}}$ was inversely associated with fibrinogen which is another acute phase protein produced in liver. An elevated fibrinogen level occurs as a short-term immune response due to chronic inflammation^46^ as well as it may increase with higher AP exposure^47^. A short to long-term AP exposure was positively associated also with interleukin-1 receptor antagonist (IL-1RA)^48^. This cytokine counter-regulates interleukin-1 beta (IL-1β) and its level was shown to be elevated in individuals with risk for cardiometabolic diseases^49^. There is also some evidence that adipose tissue is a major source for IL-1RA^50^ which may co-explain our findings of lower IL-1RA values since we presented a significant inverse correlation between $\dot{V}{\text{O}}_{{2{\text{peak}}}}$ and trunk fat mass (%). Considering also the significantly decreased pro-inflammatory TNF-α and increased anti-inflammatory IL-10 with $\dot{V}{\text{O}}_{{2{\text{peak}}}}$, these findings highlight a beneficial role of CRF which is in accordance with other findings^51^.

The adipose tissue related adipokines adiponectin and leptin were significantly associated with $\dot{V}{\text{O}}_{{2{\text{peak}}}}$. Adiponectin and leptin promote satiety and appetite, insulin secretion and sensitivity and whole-body energy homeostasis, with a primary function in the control of lipid reserves. They have been investigated mainly for obesity-related diseases^52–54^, however, there is some evidence that also AP exposure decreases adiponectin and increases leptin levels^48,55–58^. This may be a sign that AP exposure can induce a pro-inflammatory immune cell environment in blood and adipose tissue^59^. These findings were suggested to contribute to a mechanistic explanation of the relationship between AP exposure and diabetes mellitus^48^ which is however not supported by our own results. Additionally, the adiponectin-leptin ratio which is a better indicator of adipose tissue inflammation^60,61^ was not significantly different between the study regions (Tables S6, S7).

### Strengths and limitations

Major strengths of our study include a large cohort stratified according to age decades and the combined multivariate assessment of living in a high or low air polluted area of the Czech Republic, CRF, body composition, socioeconomic status and their associations with a broad spectrum of biochemical factors related to oxidative status and inflammation, which allow us to adjust the models for various possible confounders. Of note, the Moravian-Silesian region ranks among the European regions with the highest ambient AP which deserves an intensive research about air pollution related human health consequences. The life expectancy at birth in the MS and SB regions in 2021 was 75.8 and 77.4 years, respectively. This is strongly influenced by many other factors, such as socioeconomic status and other demographics, which differ between these regions, but AP may obviously play an important role. For comparisons, the life expectancy at birth in the EU and Czech republic in 2021 was 80.1 and 77.2 years, respectively^62^.

However, there are several limitations of this study. First, the cross-sectional design does not allow to reveal causality and therefore follow-up measures are intended. Also, even negligible differences may be important when the exposure is lifelong which is not covered by a cross-sectional approach. Both limitations will be solved by a further prospective longitudinal investigation. Second, one-time blood sample withdraw at the high AP site may induce acute effects in the participants. Therefore, we were not able to clearly distinguish between a chronic and acute influences of AP, particularly for oxidative status biochemical markers. Third, we showed that living in the high or low AP region of the Czech Republic is much less associated with biochemical markers of oxidative status and inflammation than age, sex, CRF or body composition. However, we do not want to downplay the importance of AP and its effect on human health. Therefore, a question of sufficient sensitivity of the investigated biochemical markers to show a higher health risk level may arise. Further research with clinical endpoints of chronic diseases is warranted. Fourth, the real AP exposure may vary during the year as well as within the regions and may be also dependent on the participants behaviour. These factors are rather difficult to control. Fifth, oxidative stress, i.e. reactive oxygen/nitrogen species and peroxides levels, were not measured directly. Finally, the study cohort is skewed towards participants with a higher education level.

## Conclusions

We did not find meaningful interactions between long-term air-pollution exposure versus markers of oxidative status and inflammation. However, we showed various significant interactions with sex, age, CRF and body composition (trunk fat mass (%)). Therefore, unlike sex and age, CRF and body composition as modifiable variables must be considered the most important factors substantially associated with the risk markers of oxidative status and inflammation. However, the significant association with the BDNF level warrants further attention.

## Methods

### Study design

The presented study is part of the multidisciplinary research project Healthy Aging in Industrial Environment—Programme 4 (4HAIE), which investigates the long-term influence of ambient air pollution on health of runners and inactive individuals^63–65^. This cross-sectional investigation focuses on biochemical markers of oxidative status, inflammation and other biochemical risk factors.

### Participants

Participants were Caucasian adults (N = 1314; age 18–65 years) who lived for at least 5 years in a high air-polluted (Moravian-Silesian) or low air-polluted (South Bohemia) region of the Czech Republic. Runners and inactive individuals were recruited from both regions with high and low air pollution. Sociodemographic characteristics of participants are in accordance with the sociodemographic characteristics of the population.

- Inclusion criteria—*runners*: running as a main exercise activity, > 150 min of moderate or > 75 min of strenuous physical activity per week (or an equivalent combination of moderate and strenuous physical activity)^66^, > 10 km running per week for at least 6 weeks prior to the tests, intending to continue running for next 12 months, permanent (> 5 years) whole-year residency in the determined areas, not planning to move away from the determined areas during the next 12 months, with internet access, using a smart phone (with iOS or Android 5.0 or higher).
- Inclusion criteria—*inactive individuals*: < 150 min of moderate or < 75 min of strenuous exercise per week, capable of running, but running irregularly and/or less than 6 weeks prior to the tests, no contraindications to exercise, permanent (> 5 years) whole-year residency in the determined areas, not planning to move away from the determined areas during the next 12 months, with internet access, using a smart phone (with iOS or Android 5.0 or higher).
- Exclusion criteria—*runners and inactive individuals*: acute (within 6 weeks) health condition (pain, injury, surgery) preventing from physical activity, any other acute disease, pregnancy, radiological examination within the last 7 days, artificial pacemaker, radioactive, surgical or any other device/implant, insulin pump, smoking.

All methods were carried out in accordance with relevant guidelines and regulations. The 4HAIE study is conducted in accordance with the Declaration of Helsinki. The study protocol has already been approved by the Ethics Committee of the University of Ostrava (3/2018). A detailed participant information sheet is provided to each individual prior to them providing written informed consent. The study poses little to no risk to participants.

### Cardiorespiratory fitness (CRF) assessment

Participants performed a graded exercise test (GXT) on a motorised treadmill (Rodby RL 2500E) to determine peak aerobic power ($\dot{V}{\text{O}}_{{2{\text{peak}}}}$). Prior to the GXT, participants completed 3 min of walking at 5.0 km/h to familiarise themselves with the treadmill. The GXT protocol then started at 6.0 km/h, with speed subsequently increasing by 1.0 km/h every minute (inclination remaining at 1%) until volitional exhaustion. Expired air was continuously monitored to analyse O_2_ and CO_2_ concentrations during the GXT with a breath-by-breath system (Blue Cherry, Geratherm Medical AG, Germany). The highest average O_2_ consumption measured over a 30 s period was used to determine $\dot{V}{\text{O}}_{{2{\text{peak}}}}$. Perceived effort was obtained using the 20-point Borg scale. All sessions were conducted in the afternoon, at least 3 h after the participants’ last meal and in a thermally controlled laboratory (21 °C, 40% relative humidity). Each participant was advised not to participate in any vigorous activity 24 h prior to the test. Participants who did not pass the Physical Activity Readiness Questionnaire (PAR-Q) were not allowed to perform the GXT unless explicit permission given by a medical doctor was provided. Blood pressure (BP) was also checked before the GXT. In case of BP values ≥ 150/90 mm Hg, participants without a medical permission were not allowed to perform the GXT, but continued in the study protocol. Detailed results for exercise performance have been presented recently^67^.

### Anthropometry

Anthropometric measurements included basic anthropometric parameters (body height and body mass) and body composition. All measurements were taken in the morning. The participants were measured in sports clothing (shorts and T-shirt) and barefoot. The standard conditions for the bioelectrical impedance analysis (BIA) method measurements were provided by the specific schedule of the study protocol. All participants were housed in a supervised accommodation at the research centre about 15 h prior to the BIA measurement which supported the standard requirements before the measurement. Body height was measured first using the InBody 370 stadiometer (Biospace, South Korea), followed by body mass and hydration status (total body water, intracellular and extracellular water), which were measured using the InBody 770 bioimpedance multifrequency scale device (Biospace, South Korea).

### Blood pressure

Systolic and diastolic blood pressure (BP) was automatically measured three times 1–2 min after the participant had been sitting for ≥ 10 min in a quiet room by applying a standard device (Nissei DM 3000, Nihon Seimitsu Sokki Co., Japan). This procedure is in line with the recommendations of the American College of Cardiology, American Heart Association, and European Society of Hypertension^68,69^. Participants were instructed to avoid caffeinated beverages for at least 60 min before the blood pressure measurements. Blood pressure greater than 140/90 excluded subjects for GXT.

### Blood analysis

Fasting blood samples were collected from the antecubital vein. Whole blood samples with EDTA as an anticoagulant were used immediately for blood count and glycated hemoglobin (HbA1c) determination and two 100-μl aliquots were frozen at − 80 °C until analysis of glutathione (GSH) and glutathione disulfide (GSSG). Whole blood samples with Li-Heparin (two 200-μl aliquots) were frozen at − 80 °C until analysis for superoxide dismutase (SOD) and glutathione peroxidase (GPx). Whole blood samples for fibrinogen analysis were collected separately in tubes with sodium citrate and centrifuged at 2500 g for 15 min to separate plasma. Serum collection tubes were allowed to clot for 30 min and subsequently centrifuged at 2500 g for 10 min to separate the serum. Blood serum was divided into five 200–500-μl aliquots, which were frozen at − 80 °C until analysis. The S-Monovette® (Sarstedt, Nümbrecht, Germany) and Vacuette^®^ systems (BD, Mississauga, Canada) were used for blood sample collection.

HbA1c was measured using a Tosoh G11 (Tosoh, Tokyo, Japan). GSH and GSSG were measured using a HPLC Prominence (Shimadzu, Kyoto, Japan). SOD and GPx were measured using an Advia 1800 (Siemen, Berlin, Germany). Glucose, triglyceride (TG), total cholesterol, high- and low-density lipoprotein cholesterol (HDL-C and LDL-C, respectively), and C-reactive protein (CRP) concentrations were measured using a Cobas 8000 device (Roche, Basel, Switzerland).

Cytokines and growth factor determinations were performed in a series of runs with different batches of kits so that the expiration of the kits was not exceeded. This analysis was performed by the ALBIA (addressable laser bead assay) technique and the tests performed were measured and evaluated on a Luminex 200. The following R&D Systems kits supplied by Bio-Techne R&D Systems were used: LXSAHM-01 Adiponectin, LXSAHM-01 brain-derived neurotrophic factor (BDNF) and LXSAHM-06 interleukin-1β (IL-1β), interleukin-1 receptor antagonist (IL-1RA), high-sensitive interleukin-6 (hs-IL-6), interleukin-10 (IL-10), tumor necrosis factor alpha (TNFα) and leptin.

The intra-assay coefficients of variation for biochemical and blood count parameters were < 5%. Leptin, adiponectin, TNF-α, IL-1RA, IL-1β, IL-10, and hs-IL-6 were determined with an inter-assay coefficient lower than 10%.

### Statistical analysis

As part of the preprocessing, null values that corresponded to measurements below the detection limit were imputed 1/2 the detection limit. All statistical analyses were performed with the maximum available data using the R software (version 4.0.2, www.r-project.org) and the significance level was set to 0.05.

In the first step, the basic characteristics of the cohort and the descriptive characteristics of the variables used were calculated. Categorical variables are presented as absolute frequencies and relative frequencies in percentages. For a univariate description of numerical variables, the minimum (Min), the maximum (Max), the median (M) and the interquartile range (IQR) were used because of the non-normality of the data (tested by the Shapiro–Wilk test).

Second, multiple regression analysis was used to explain the relationship between multiple independent variables ($\dot{V}{\text{O}}_{{2{\text{peak}}}}$, trunk fat mass, sex (male vs. female), socioeconomic status represented as an achieved education level (1—basic school, including unfinished; 2—apprentice, secondary vocational without GCSE, secondary school diploma, higher vocational; 3—university), and region (MS region vs. SB region) and dependent variables (oxidative status, inflammation, and other biochemical markers). Because of the outliers detection and the assumptions for linear regression, such as normality of the residuals, were violated, quantile (median) regression models were used. Due to a significant linear association (collinearity) between $\dot{V}{\text{O}}_{{2{\text{peak}}}}$ and trunk fat mass (Pearson’s ρ (95% CI): − 0.813 (− 0.832, − 0.793)) two models were designed for each dependent variable. Model 1 with $\dot{V}{\text{O}}_{{2{\text{peak}}}}$, sex, socioeconomic status (SES) and region as independent variables and an alternative Model 2 with trunk fat mass instead of $\dot{V}{\text{O}}_{{2{\text{peak}}}}$ for sensitivity analysis. In tables with results of regression models, asterisks (*p < .05; **p < .01) indicate which beta weights are significant.

### Supplementary Information

Supplementary Information.

## Data Availability

The raw data supporting the conclusions of this article will be available upon request pending application and approval from a corresponding author, without undue reservation.

## References

[1] World Health Organization. *WHO Global Air Quality Guidelines: Particulate Matter (PM2.5 and PM10), Ozone, Nitrogen Dioxide, Sulfur Dioxide and Carbon Monoxide. Executive Summary*. https://apps.who.int/iris/bitstream/handle/10665/345334/9789240034433-eng.pdf (2021). 34662007

[2] World Health Organization. *Ambient (Outdoor) Air Pollution*. https://www.who.int/news-room/fact-sheets/detail/ambient-(outdoor)-air-quality-and-health (2021).

[3] Khomenko S (2021). Premature mortality due to air pollution in European cities: A health impact assessment. Lancet Planet. Health.

[4] Juginović A, Vuković M, Aranza I, Biloš V (2021). Health impacts of air pollution exposure from 1990 to 2019 in 43 European countries. Sci. Rep..

[5] Brook RD, Newby DE, Rajagopalan S (2018). Air pollution and cardiometabolic disease: An update and call for clinical trials. Am. J. Hypertens..

[6] Thurston GD (2017). A joint ERS/ATS policy statement: What constitutes an adverse health effect of air pollution? An analytical framework. Eur. Respir. J..

[7] Shepherd A, Mullins JT (2019). Arthritis diagnosis and early-life exposure to air pollution. Environ. Pollut..

[8] Yang Y (2019). Short-term and long-term exposures to fine particulate matter constituents and health: A systematic review and meta-analysis. Environ. Pollut..

[9] Newby DE (2015). Expert position paper on air pollution and cardiovascular disease. Eur. Heart J..

[10] Yang, D., Yang, X., Deng, F. & Guo, X. Ambient air pollution and biomarkers of health effect. In *Advances in Experimental Medicine and Biology*, Vol. 1017 59–102 (Springer, 2017). 10.1007/978-981-10-5657-4_4 29177959

[11] Bai Y, Sun Q (2016). Fine particulate matter air pollution and atherosclerosis: Mechanistic insights. Biochim. Biophys. Acta.

[12] Marchini T, Zirlik A, Wolf D (2020). Pathogenic role of air pollution particulate matter in cardiometabolic disease: Evidence from mice and humans. Antioxid. Redox Signal..

[13] Franklin BA, Brook R, Arden Pope C (2015). Air pollution and cardiovascular disease. Curr. Probl. Cardiol..

[14] Garber CE (2011). American College of Sports Medicine position stand. Quantity and quality of exercise for developing and maintaining cardiorespiratory, musculoskeletal, and neuromotor fitness in apparently healthy adults: guidance for prescribing exercise. Med. Sci. Sports Exerc..

[15] Tainio M (2016). Can air pollution negate the health benefits of cycling and walking?. J. Transp. Health..

[16] Qin F (2019). Exercise and air pollutants exposure: A systematic review and meta-analysis. Life Sci..

[17] Daigle CC (2003). Ultrafine particle deposition in humans during rest and exercise. Inhal. Toxicol..

[18] Pierson WE, Covert DS, Koenig JQ, Namekata T, Kim YS (1986). Review: Implications of air pollution effects on athletic performance. Atmos. Environ..

[19] Bos I (2013). Subclinical effects of aerobic training in urban environment. Med. Sci. Sports Exerc..

[20] Blair C, Walls J, Davies NW, Jacobson GA (2010). Volatile organic compounds in runners near a roadway: Increased blood levels after short-duration exercise. Br. J. Sports Med..

[21] Cavalcante De Sá M (2016). Aerobic exercise in polluted urban environments: Effects on airway defense mechanisms in young healthy amateur runners. J. Breath Res..

[22] Liu Q (2022). Long-term exposure to fine particulate matter modifies the association between physical activity and hypertension incidence. J. Sport Health Sci..

[23] D’Oliveira A (2023). Impact of air pollution on the health of the older adults during physical activity and sedentary behavior: A systematic review. Environ. Res..

[24] Prada D, López G, Solleiro-Villavicencio H, Garcia-Cuellar C, Baccarelli AA (2020). Molecular and cellular mechanisms linking air pollution and bone damage. Environ. Res..

[25] Fadadu RP, Abuabara K, Balmes JR, Hanifin JM, Wei ML (2023). Air pollution and atopic dermatitis, from molecular mechanisms to population-level evidence: A review. Int. J. Environ. Res. Public Health.

[26] Shukla A (2019). Air pollution associated epigenetic modifications: Transgenerational inheritance and underlying molecular mechanisms. Sci. Total Environ..

[27] Esposito S (2014). Possible molecular mechanisms linking air pollution and asthma in children. BMC Pulm. Med..

[28] Zhang H (2020). Ambient air pollution and gestational diabetes mellitus: A review of evidence from biological mechanisms to population epidemiology. Sci. Total Environ..

[29] Liu Y, Goodson JM, Zhang B, Chin MT (2015). Air pollution and adverse cardiac remodeling: clinical effects and basic mechanisms. Front. Physiol..

[30] You Y (2022). Physical exercise in the context of air pollution: An emerging research topic. Front. Physiol..

[31] Michalik J, Machaczka O, Jirik V, Heryan T, Janout V (2022). Air pollutants over industrial and non-industrial areas: Historical concentration estimates. Atmosphere (Basel).

[32] CHMU. Air Pollution in the Czech Republic 2020. https://info.chmi.cz/rocenka/ko2020/7.php (2020).

[33] Pei J, Pan X, Wei G, Hua Y (2023). Research progress of glutathione peroxidase family (GPX) in redoxidation. Front. Pharmacol..

[34] Alkazemi D, Rahman A, Habra B (2021). Alterations in glutathione redox homeostasis among adolescents with obesity and anemia. Sci. Rep..

[35] Mudway IS, Kelly FJ, Holgate ST (2020). Oxidative stress in air pollution research. Free Radic. Biol. Med..

[36] Calabró V (2021). Urban air pollution induces alterations in redox metabolism and mitochondrial dysfunction in mice brain cortex. Arch. Biochem. Biophys..

[37] Zuccato C, Cattaneo E (2009). Brain-derived neurotrophic factor in neurodegenerative diseases. Nat. Rev. Neurol..

[38] Cavaleri D (2023). The role of BDNF in major depressive disorder, related clinical features, and antidepressant treatment: Insight from meta-analyses. Neurosci. Biobehav. Rev..

[39] de Lima NS (2022). Moderate-intensity continuous training and high-intensity interval training improve cognition, and BDNF levels of middle-aged overweight men. Metab. Brain Dis..

[40] Bos I, De Boever P, Int Panis L, Meeusen R (2014). Physical activity, air pollution and the brain. Sport. Med..

[41] Silveira AC (2022). Effects of air pollution exposure on inflammatory and endurance performance in recreationally trained cyclists adapted to traffic-related air pollution. Am. J. Physiol. Integr. Comp. Physiol..

[42] Dix C (2022). C-reactive protein, immunothrombosis and venous thromboembolism. Front. Immunol..

[43] Liu Q (2019). Ambient particulate air pollution and circulating C-reactive protein level: A systematic review and meta-analysis. Int. J. Hyg. Environ. Health.

[44] Kjerulff B (2023). Medium term moderate to low-level air pollution exposure is associated with higher C-reactive protein among healthy Danish blood donors. Environ. Res..

[45] Mac Giollabhui N (2020). To exclude or not to exclude: Considerations and recommendations for C-reactive protein values higher than 10 mg/L. Brain. Behav. Immun..

[46] Davalos D, Akassoglou K (2012). Fibrinogen as a key regulator of inflammation in disease. Semin. Immunopathol..

[47] Hampel R (2015). Long-term effects of elemental composition of particulate matter on inflammatory blood markers in European cohorts. Environ. Int..

[48] Lucht S (2019). Air pollution and diabetes-related biomarkers in non-diabetic adults: A pathway to impaired glucose metabolism?. Environ. Int..

[49] Herder C (2017). Circulating levels of interleukin 1-receptor antagonist and risk of cardiovascular disease. Arterioscler. Thromb. Vasc. Biol..

[50] Juge-Aubry CE (2003). Adipose tissue is a major source of interleukin-1 receptor antagonist: Upregulation in obesity and inflammation. Diabetes.

[51] Wedell-Neergaard A-S (2018). Cardiorespiratory fitness and the metabolic syndrome: Roles of inflammation and abdominal obesity. PLoS One.

[52] Picó C, Palou M, Pomar CA, Rodríguez AM, Palou A (2022). Leptin as a key regulator of the adipose organ. Rev. Endocr. Metab. Disord..

[53] Saxton RA (2023). Structural insights into the mechanism of leptin receptor activation. Nat. Commun..

[54] Lelis DDF, de Freitas DF, Machado AS, Crespo TS, Santos SHS (2019). Angiotensin-(1–7), adipokines and inflammation. Metabolism.

[55] Wang Y (2014). Long-term exposure to ambient air pollution and serum leptin in older adults. J. Occup. Environ. Med..

[56] Campolim CM (2020). Short-term exposure to air pollution (PM2.5) induces hypothalamic inflammation, and long-term leads to leptin resistance and obesity via Tlr4/Ikbke in mice. Sci. Rep..

[57] Haberzettl P (2021). Fine particulate matter air pollution and aortic perivascular adipose tissue: Oxidative stress, leptin, and vascular dysfunction. Physiol. Rep..

[58] Shi X, Zheng Y, Cui H, Zhang Y, Jiang M (2022). Exposure to outdoor and indoor air pollution and risk of overweight and obesity across different life periods: A review. Ecotoxicol. Environ. Saf..

[59] Rahman MM (2022). Near-roadway air pollution, immune cells and adipokines among obese young adults. Environ. Health.

[60] Frühbeck G (2019). Adiponectin-leptin ratio is a functional biomarker of adipose tissue inflammation. Nutrients.

[61] Cipryan L, Dostal T, Plews DJ, Hofmann P, Laursen PB (2021). Adiponectin/leptin ratio increases after a 12-week very low-carbohydrate, high-fat diet, and exercise training in healthy individuals: A non-randomized, parallel design study. Nutr. Res..

[62] Eurostat. Life expectancy at birth down to 80.1 years in 2021. https://ec.europa.eu/eurostat/web/products-eurostat-news/w/DDN-20230316-1 (2023).

[63] Cipryan L (2020). Regular running in an air-polluted environment: Physiological and anthropometric protocol for a prospective cohort study (Healthy Aging in Industrial Environment Study—Program 4). BMJ Open.

[64] Elavsky S (2021). Physical activity in an air-polluted environment: Behavioral, psychological and neuroimaging protocol for a prospective cohort study (Healthy Aging in Industrial Environment study—Program 4). BMC Public Health.

[65] Jandacka D (2020). Running and physical activity in an air-polluted environment: The biomechanical and musculoskeletal protocol for a prospective cohort study 4HAIE (Healthy Aging in Industrial Environment—Program 4). Int. J. Environ. Res. Public Health.

[66] *Guidelines for Exercise Testing and Prescription (ACSM)*. (Wolters Kluwer Health | Lippincott Williams & Wilkins, 2018).

[67] Birnbaumer P, Dostal T, Cipryan L, Hofmann P (2023). Pattern of the heart rate performance curve in maximal graded treadmill running from 1100 healthy 18–65 Years old men and women: The 4HAIE study. Front. Physiol..

[68] Casey DE (2019). 2019 AHA/ACC clinical performance and quality measures for adults with high blood pressure: A report of the American College Of Cardiology/American Heart Association task force on performance measures. Circ. Cardiovasc. Qual. Outcomes.

[69] Parati G (2008). European Society of Hypertension guidelines for blood pressure monitoring at home: a summary report of the Second International Consensus Conference on Home Blood Pressure Monitoring. J. Hypertens..

