# Supplementary material for: Air pollution, cardiorespiratory fitness and biomarkers of oxidative status and inflammation in the 4HAIE study
Source: Sci Rep. 2024 Apr 26;14:9620. doi: 10.1038/s41598-024-60388-w (PMC11053001; doi:10.1038/s41598-024-60388-w)
Supplement: Supplementary file 1 — Supplementary Information. [file 41598_2024_60388_MOESM1_ESM.pdf]

Living in air polluted regions - Association between cardiorespiratory fitness, oxidative status and inflammatory biomarkers: A multivariate regression analysis of the HAIE study.

Lukas Cipryan[1]*, Martina Litschmannova[2], Tomas Barot[3], Tomas Dostal[1], Dominik Sindler[1], Petr Kutac[1], Daniel Jandacka[1], and Peter Hofmann[4]

Supplemental Material

Table S1. Basic characteristics of the cohort (Model 2).

Table S2. Descriptive characteristics of oxidative status, inflammation and other biochemical markers (Model 2).

Table S3. A multi-regression analyses of the oxidative status markers (Model 2).

Table S4. A multi-regression analyses of the inflammation markers (Model 2).

Table S5. A multi-regression analyses of the inflammation and other biochemical markers (Model 2).

Table S6. Descriptive characteristics of oxidative status, inflammation and other biochemical markers in the Moravian-Silesian Region (N = 663) and South Bohemia Region (N = 525).

Table S7. Descriptive characteristics of oxidative status, inflammation and other biochemical markers in the Moravian-Silesian Region (N = 663) and South Bohemia Region (N = 525).

Table S1. Basic characteristics of the cohort (Model 2; N = 1,300).

Males / Females	700 (54%) / 600 (46%)	
MS / SB Region	744 (57%) / 556 (43%)	
SES – 1	63 (5%)	
SES – 2	672 (52%)	
SES – 3	565 (43%)	
	(Min; Max)	M (IQR)
Age (years)	(18.0; 65.0)	39.0 (27.0; 47.0)
Height (cm)	(148.5; 201.6)	174.5 (167.9; 181.2)
Body mass (kg)	(40.5; 127.4)	74.2 (64.4; 84.9)
Total body fat (%)	(3.5; 52.9)	21.0 (15.5; 28.2)
Trunk fat mass (%)	(14.1; 51.9)	27.3 (22.4; 33.1)
$\dot{V}O_{2peak}$ (ml/kg/min)	(14.6; 70.9)	41.7 (34.6; 49.2)
Systolic BP (mmHg)	(84.0; 180.0)	127.0 (117.0; 137.0)
Diastolic BP (mmHg)	(45.3; 111.7)	79.00 (72.3; 86.3)
Glucose (mmol/l)	(1.65; 8.06)	4.76 (4.48; 5.08)
HbA1c (mmol/l)	(9.0; 63.0)	35.0 (32.0; 37.0)
Total cholesterol (mmol/l)	(1.50; 8.90)	2.90 (2.40; 3.70)
HDL-CH (mmol/l)	(0.68; 3.80)	1.56 (1.27; 1.86)
LDL-CH (mmol/l)	(0.47; 6.51)	2.81 (2.30; 3.47)
Triglycerides (mmol/l)	(0.18; 6.11)	1.00 (0.74; 1.37)
Free fatty acids (mmol/l)	(0.06; 1.44)	0.40 (0.29; 0.56)

Legend: SB – South Bohemia Region (low air polluted area), MS – Moravian-Silesian Region (high air polluted area); $\dot{V}O_{2peak}$ – peak oxygen consumption; BP – blood pressure; HbA1c – glycated hemoglobin; HDL-CH/LDL-CH – high/low density lipoprotein cholesterol; SES – socioeconomic status represented as an achieved education level (see Methods).
M – median; IQR – interquartile range

Table S2. Descriptive characteristics of oxidative status, inflammation and other biochemical markers (Model 2; N= 1,300).

	(Min; Max)	M (IQR)
SOD (U/ml)	(123; 529)	237 (216; 260)
GPx (U/l)	(2,852; 27,393)	11,233 (9,628; 12,882)
GSSG (μmol/l)	(6.0; 228.0)	72.0 (50.0; 97.0)
GSH (μmol/l)	(374; 2610)	1107 (962; 1246)
GSH/GSSG (-)	(2.6; 225.5)	15.6 (10.5; 23.8)
IL-1β (pg/ml)	(0.01; 73.00)	0.39 (0.19; 0.51)
IL-1RA (pg/ml)	(0; 6,235)	467 (346 ; 624)
hs-IL-6 (pg/ml)	(0.00; 145.77)	0.18 (0.07; 0.54)
IL-10 (pg/ml)	(0.01; 64.47)	0.12 (0.05; 0.41)
TNF-α (pg/ml)	(0.02; 10.89)	0.91 (0.39; 1.58)
CRP (mg/l)	(0.50; 55.10)	0.50 (0.50; 1.70)
Fibrinogen (g/l)	(0.45; 5.00)	2.62 (2.31; 2.97)
Adiponectin (μg/ml)	(0.00; 106.19)	5.94 (3.70; 8.95)
Leptin (ng/ml)	(0.00; 93.11)	6.24 (2.47; 12.50)
Adpn/Lep (-)	(0.00; 8,570.14)	0.96 (0.40; 2.54)
BDNF (ng/ml)	(0.0; 142.8)	24.3 (19.1; 29.7)

Legend: SOD – superoxide dismutase; GPx – glutathione peroxidase; GSSG – glutathione disulphide; GSH – glutathione; IL-1β – interleukin 1β; IL-1RA – interleukin-1 receptor antagonist; hs-IL-6 – high-sensitive interleukin 6. IL-10 – interleukin 10; TNF-α – tumor necrosis factor alpha; CRP – C-reactive protein; Adpn/Lep – adiponectin-leptin ratio; BDNF – brain-derived neurotrophic factor.
M – median; IQR – interquartile range.

Table S3. A multi-regression analyses of the oxidative status markers (Model 2).

	SOD	GPx	GSSG	GSH	GSH/GSSG
(Intercept)	196.371**	9,802.856**	63.699**	1,128.690**	16.984**
Sex_male	38.671**	142.533	-0.534	45.438*	1.005
SES 2-4	1.406	416.357	3.468	0.350	-2.006
SES 5	-3.600	386.621	2.701	-12.538	-2.825
MS Region	0.921	282.985	-1.485	-9.036	0.420
Age	-0.348**	14.918*	0.193	-0.003	-0.033
Trunk fat mass	1.212**	5.685	-0.044	-1.431	0.049

Legend: SOD – superoxide dismutase; GPx – glutathione peroxidase; GSSG – glutathione disulphide; GSH – glutathione.

SES – socioeconomic status represented as an achieved education level (see Methods); MS – Moravian-Silesian Region; $\dot{V}O_{2peak}$ – peak oxygen consumption.

* $p < .05$; ** $p < .01$

Table S4. A multi-regression analyses of the inflammation markers (Model 2).

	IL-1β	IL-1RA	hs-IL-6	IL-10	TNF-α
(Intercept)	0.398**	163.301**	-0.021	0.130**	0.531**
Sex_male	-0.004	15.130	0.004	0.006	0.222**
SES 2-4	0.127**	1.973	0.107	0.011	-0.167
SES 5	0.121*	-7.564	-0.007	0.006	-0.204
MS Region	-0.002	-23.992	0.013	0.012	-0.020
Age	0.001	-0.555	0.003**	0.001**	0.002
Trunk fat mass	-0.006**	12.275**	0.003	-0.002**	0.013**

Legend: IL-1β – interleukin 1β; IL-1RA – interleukin-1 receptor agonist; hs-IL-6 – high-sensitive interleukin 6; IL-10 – interleukin 10; TNF-α – tumor necrosis factor alpha.
SES – socioeconomic status represented as an achieved education level (see Methods); MS – Moravian-Silesian Region; $\dot{V}O_{2peak}$ – peak oxygen consumption.
* p < .05; ** p < .01

Table S5. A multi-regression analyses of the inflammation and other biochemical markers (Model 2).

	CRP	Fibrinogen	Adiponectin	Leptin	Adpn/Lep	BDNF
(Intercept)	-0.710**	1.850**	8,495.689**	-6,822.889**	3.551**	21,239.369**
Sex_male	0.134**	-0.069*	-1,721.574**	-3,330.599**	0.239**	-70.412
SES 2-4	-0.075	-0.085	517.622	-474.016	0.193	-149.867
SES 5	-0.173	-0.165*	525.169	-929.776	0.177	-361.278
MS Region	-0.052	-0.045	-191.148	-602.612**	0.105	-1,440.687*
Age	-0.000	0.006**	29.434**	-72.546**	0.014**	3.625
Trunk fat mass	0.062**	0.025**	-108.523**	743.413**	-0.111**	137.417**

Legend: CRP – C-reactive protein, BDNF – brain-derived neurotrophic factor.
SES – socioeconomic status represented as an achieved education level (see Methods); MS – Moravian-Silesian Region; $\dot{V}O_{2peak}$ – peak oxygen consumption.
* p < .05; ** p < .01

Table S6. Descriptive characteristics of oxidative status, inflammation and other biochemical markers in the Moravian-Silesian Region (N = 663) and South Bohemia Region (N = 525) for Model 1.

	Moravian-Silesian Region	South Bohemia Region
	M (IQR)	M (IQR)
Age (years)	38.0 (28.0; 47.0)	37.0 (25.0; 45.0)
Height (cm)	173.9 (167.7; 181.2)	174.8 (168.2; 181.7)
Body mass (kg)	74.0 (64.0; 84.3)	73.8 (63.3; 84.2)
Total body fat (%)	20.6 (15.5; 28.0)	20.7 (14.6; 27.2)
Trunk fat mass (%)	27.0 (22.4; 33.1)	26.8 (22.0; 32.1)
$\dot{V}O_{2peak}$ (ml/kg/min)	41.6 (34.1; 48.6)	42.0 (34.9; 49.5)
Systolic BP (mmHg)	126.0 (116.0; 134.0)	125.0 (116.3; 134.0)
Diastolic BP (mmHg)	78.3 (72.0; 85.0)	77.7 (71.3; 84.0)
Glucose (mmol/l)	4.78 (4.49; 5.08)	4.71 (4.46; 5.01)
HbA1c (mmol/l)	35.0 (32.0; 37.0)	35.0 (32.0; 37.0)
Total cholesterol (mmol/l)	2.90 (2.40; 3.70)	2.90 (2.50; 3.70)
HDL-CH (mmol/l)	1.56 (1.26; 1.86)	1.55 (1.28; 1.87)
LDL-CH (mmol/l)	2.76 (2.25; 3.40)	2.79 (2.28; 3.45)
Triglycerides (mmol/l)	0.96 (0.72; 1.30)	1.01 (0.75; 1.36)
Free fatty acids (mmol/l)	0.40 (0.29; 0.56)	0.40 (0.27; 0.58)

Legend: Moravian-Silesian Region (high air polluted area); South Bohemia Region (low air polluted area); $\dot{V}O_{2peak}$ – peak oxygen consumption; BP – blood pressure; HbA1c – glycated hemoglobin; HDL-CH/LDL-CH – high/low density lipoprotein cholesterol; SES – socioeconomic status represented as an achieved education level (see Methods). M – median; IQR – interquartile range

Table S7. Descriptive characteristics of oxidative status, inflammation and other biochemical markers in the Moravian-Silesian Region (N = 663) and South Bohemia Region (N = 525) for Model 1.

	Moravian-Silesian Region	South Bohemia Region
	M (IQR)	M (IQR)
SOD (U/ml)	237.00 (213.5; 259.5)	237.0 (216.0; 260.0)
GPx (U/l)	11,348 (9,579; 12,966)	11,000 (9,629; 12,801)
GSSG (µmol/l)	72.0 (51.0; 95.0)	72.0 (50.0; 99.0)
GSH (µmol/l)	1,097 (961; 1,231)	1,114 (958; 1,272)
GSH/GSSG (-)	15.4 (10.8; 23.7)	15.6 (10.2; 23.9)
IL-1β (pg/ml)	0.39 (0.22; 0.51)	0.39 (0.17; 0.51)
IL-1RA (pg/ml)	469 (344; 620)	480 (359; 629)
hs-IL-6 (pg/ml)	0.18 (0.09; 0.57)	0.15 (0.07; 0.47)
IL-10 (pg/ml)	0.12 (0.06; 0.42)	0.12 (0.05; 0.40)
TNF-α (pg/ml)	0.91 (0.39; 1.60)	0.90 (0.38; 1.48)
CRP (mg/l)	0.50 (0.50; 1.60)	0.50 (0.50; 1.90)
Fibrinogen (g/l)	2.58 (2.27; 2.97)	2.62 (2.31; 2.94)
Adiponectin (µg/ml)	5.91 (3.75; 8.50)	6.05 (3.73; 9.37)
Leptin (ng/ml)	5.53 (2.45; 11.68)	6.49 (2.30; 12.71)
Adpn/Lep (-)	1.06 (0.42; 2.60)	0.90 (0.38; 2.52)
BDNF (ng/ml)	23.7 (18.6; 29.3)	24.8 (19.5; 30.3)

Legend: SOD – superoxide dismutase; GPx – glutathione peroxidase; GSSG – glutathione disulphide; GSH – glutathione; IL-1β – interleukin 1β; IL-1RA – interleukin-1 receptor antagonist; hs-IL-6 – high-sensitive interleukin 6. IL-10 – interleukin 10; TNF-α – tumor necrosis factor alpha; CRP – C-reactive protein; Adpn/Lep – adiponectin-leptin ratio; BDNF – brain-derived neurotrophic factor.
M – median; IQR – interquartile range.
